# Applying Deep Learning to Image and IMU Sensor Data for Real-Time Fatigue Recognition in Table Tennis Players

**DOI:** 10.3390/s26206390

**Published:** 2026-10-09

**Authors:** Yung-Hoh Sheu, Tzu-Hsuan Tai, Tz-Yun Chen, Cheng-Yu Huang, Sheng K. Wu

**Affiliations:** 1 Department of Computer Science and Information Engineering, National Formosa University, Yunlin 632, Taiwan; yhsheu@nfu.edu.tw (Y.-H.S.); 40943247@gm.nfu.edu.tw (T.-H.T.); andy91087@gmail.com (C.-Y.H.); 2 Office of Physical Education, National Formosa University, Yunlin 632, Taiwan; yvonne@nfu.edu.tw; 3 Department of Kinesiology, National Tsing Hua University, Hsinchu 300, Taiwan

**Keywords:** neural networks, fatigue recognition, nine-axis IMU sensor, vision-based analysis, multimodal sensing

## Abstract

Fatigue during table tennis training can impair performance and increase the risk of injury. This study proposes a real-time fatigue recognition system that integrates an embedded edge-AI racket with a camera for synchronized multimodal data acquisition. On the racket side, a neural network was deployed on an edge-AI microcontroller unit (MCU) to enable on-device inference, while the test data were transmitted to a computer for multimodal feature fusion. Given the continuous nature of fatigue development, the data were divided into three stages: non-fatigue, transition, and fatigue to facilitate progressive evaluation. In the binary classification task, which distinguished between the two extreme states, the multimodal hybrid model achieved an accuracy of 99%. When the transition stage was incorporated into a three-class classification task, performance declined because of the ambiguous boundaries between fatigue states. However, the use of multimodal decision-fusion strategies, such as weighted soft voting, maintained an accuracy of 96%. Overall, the results demonstrate the feasibility of deep learning for real-time fatigue recognition during table tennis training and provide a practical monitoring tool to help players track their physiological states and reduce injury risk.

## 1. Introduction

Table tennis is a physically demanding sport that requires repeated strokes and rapid recovery, and prolonged high-intensity training can induce muscle fatigue. Under fatigue, the neuromuscular system tends to maintain performance through compensatory movement patterns, which may reduce stroke quality and increase the risk of sports injury [1,2,3]. Conventional assessment has relied on coaches’ subjective observation and lacks quantitative feedback; however, advances in sensing and computing technologies now allow wearable IMUs and computer vision to capture movement and biomechanical information in a complementary, real-time manner, driving sports science toward a more quantitative, data-driven approach.

The bandwidth and latency limitations of cloud-computing architectures make it difficult to meet the real-time feedback requirements of table tennis. Edge AI can deploy optimized neural network models on sensor-side microcontroller units (MCUs), allowing devices to operate offline and provide continuous real-time feedback [4]. Existing studies are largely limited to a single sensing modality, and early fatigue-assessment approaches relied on invasive measurements or subjective scales that are difficult to monitor in real time; research has therefore shifted toward human activity recognition methods that combine wearable IMU (Inertial Measurement Unit) with deep learning, using CNNs for feature extraction and LSTMs to mitigate the vanishing-gradient problem in long sequences [5]. Vision-only or single-modality approaches remain constrained by ambiguity between similar postures, occlusion, motion blur, and information gaps.

IMUs have been widely integrated into smart racquet-sport equipment owing to their portability and relatively low cost and have been used for accurate stroke classification [6], proving valuable for motion analysis in sports medicine more broadly [7]. However, a single sensor’s local scope and integration drift make it difficult to reconstruct whole-body posture or identify the source of cross-segment compensation. In table tennis specifically, lower-limb footwork substantially influences upper-limb stroke quality: when lower-limb control is insufficient or the body’s center of mass becomes unstable, racket control deteriorates and stroke performance declines [8]. Because a wrist-mounted IMU alone lacks cross-limb spatial context, it cannot distinguish whether an anomalous wrist vibration originates from local muscular fatigue or from lower-limb compensatory adjustment. Multimodal fusion has become an important direction in human activity recognition: decision-level fusion is better able to handle alignment issues arising from heterogeneous, asynchronously sampled data, and soft voting aggregates probability distributions to reduce sensitivity to noise [9]. This study adopts weighted soft voting, which assigns each sub-model an adjustable reliability weight to improve accuracy [10].

Based on this background, the objective of this study is to develop a real-time fatigue-recognition method for table tennis that integrates IMU, computer vision, and edge AI: combining multimodal features to address information gaps, introducing weighted soft voting to handle ambiguous features during the transition stage, and designing an embedded AI table tennis racket that performs low-latency on-device inference while preserving a natural grip feel. These components are integrated into a real-time fatigue-monitoring system intended to shift training from an experience-based to a data-driven approach. In this context, real-time is defined relative to the timescale of the physiological process being monitored and the experimental protocol’s decision cadence, rather than sub-second control-loop latency: the system is designed to deliver updated fatigue assessments well within the interval between successive changes in training load.

## 2. Materials and Methods

### 2.1. System Overview

**Figure 1:**
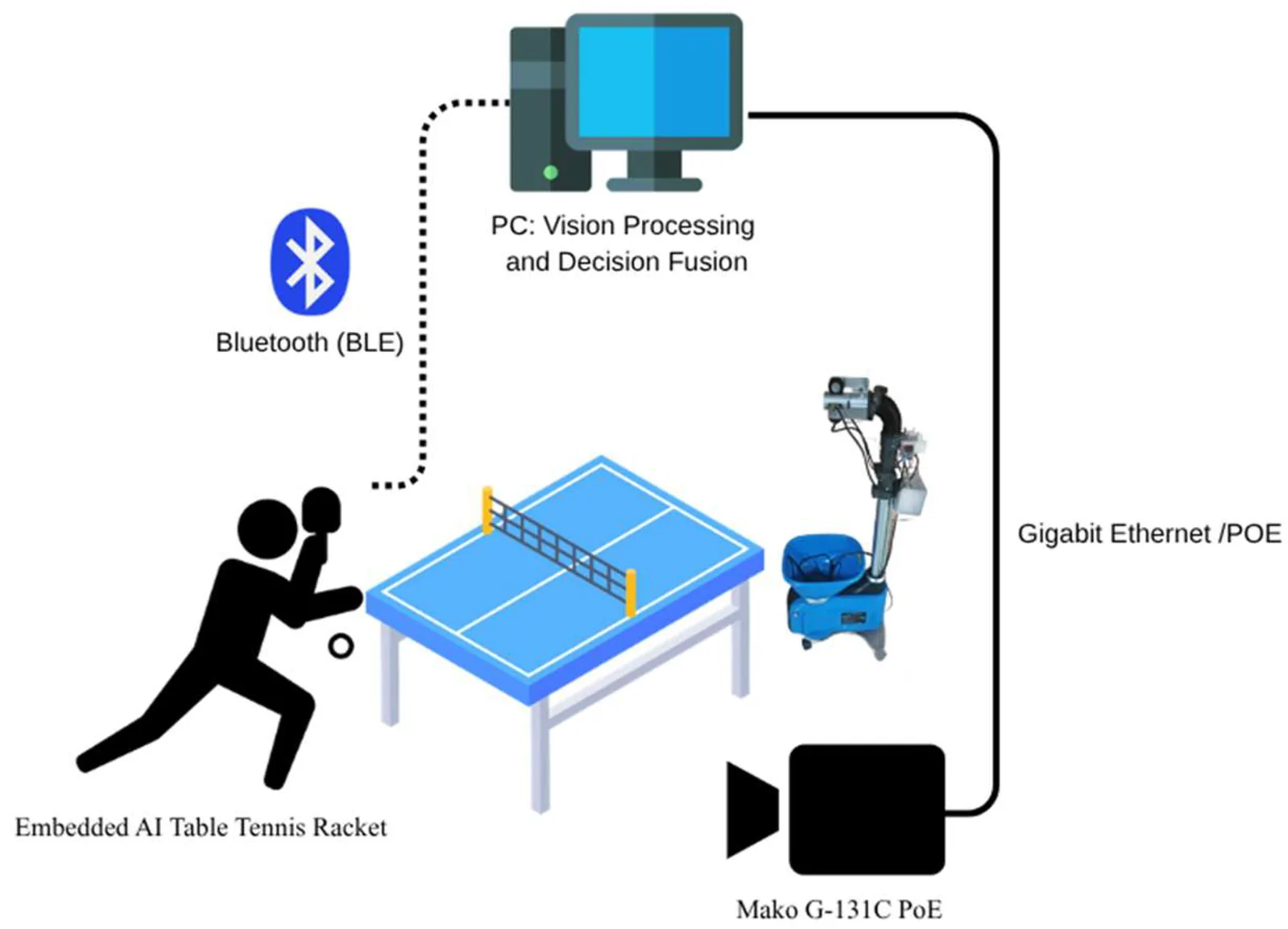
System context diagram showing data flow among the embedded AI table tennis racket, the high-speed camera, and the host computer.

The system integrates an embedded AI table tennis racket built around a nine-axis IMU (ICM-20948, TDK InvenSense, Japan) and an STM32F7-series MCU (STMicroelectronics, Switzerland), paired with a high-speed camera to synchronously collect data during aerobic exercise testing. Figure 1 shows a schematic diagram of the overall system architecture and context. On the racket side, inertial feature sampling and inference are completed on-device, after which the results and feature data are transmitted to the host computer via a Bluetooth Low Energy (BLE) module (HM-19, Jinan Huamao Technology, China). The high-speed camera simultaneously captures the stroke motion and streams the video to the host computer over Ethernet. The host computer performs pose estimation (Sports2D v.0.8.31) and biomechanical conversion (OpenSim v.4.6) on the video stream, temporally aligns the resulting features with the racket-side IMU features and inference results, and finally applies the multimodal model and weighted soft voting to produce the final fatigue classification result.

**Figure 2:**
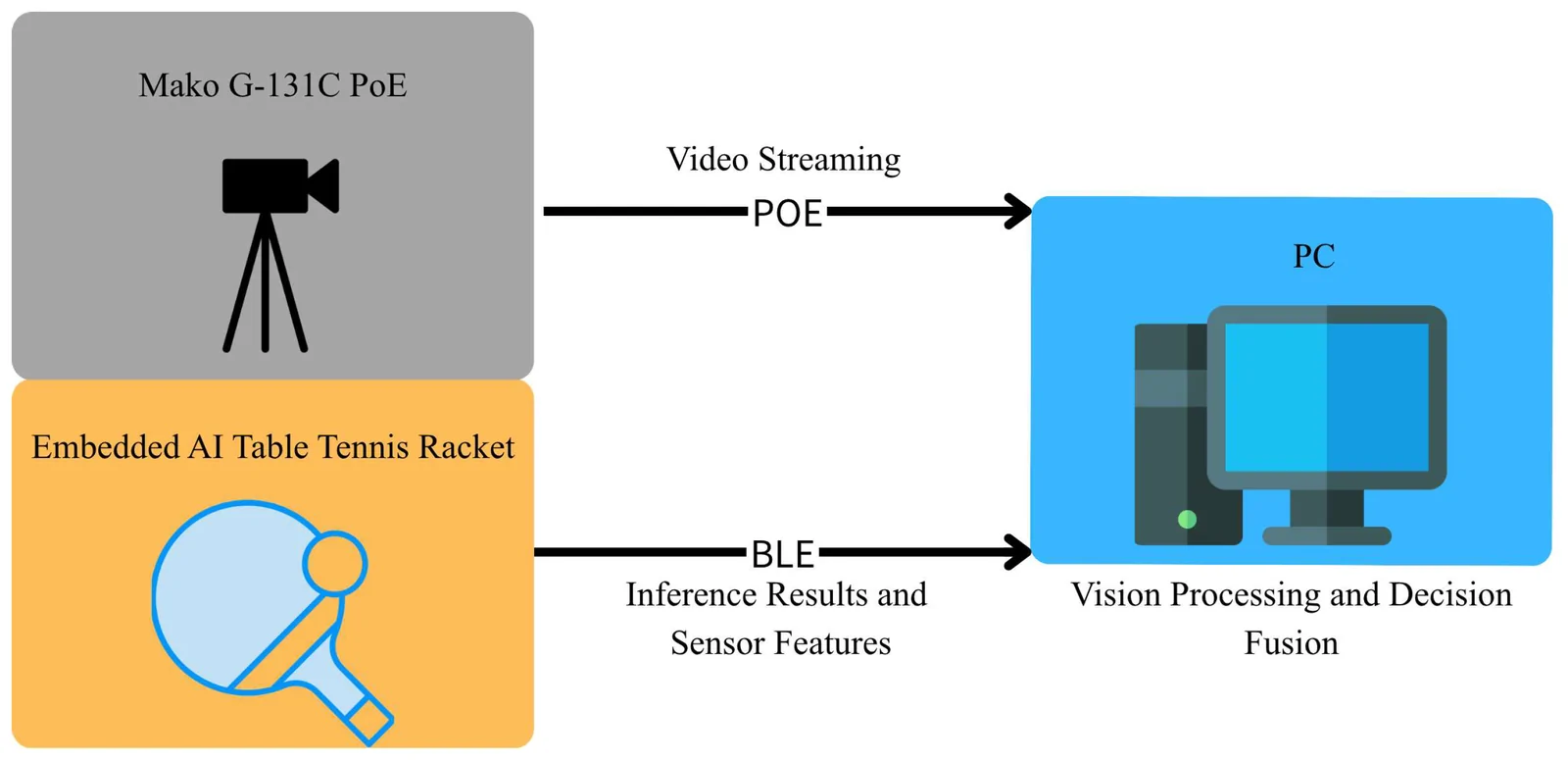
Overall system architecture. The host computer acts as the master controller, coordinating synchronized data acquisition from the racket-embedded IMU and the camera and performing multimodal fusion.

The overall system architecture is shown in Figure 2. The system adopts a master–slave control architecture, in which the host computer acts as the master, responsible for initiating data collection, controlling the connected devices, and managing time synchronization. At the start of a test, the host computer issues a data-collection command, triggering the racket-embedded IMU and the camera to acquire data synchronously. The fatigue-recognition models are deployed separately on the racket side (edge inference) and on the host computer (multimodal fusion), forming a distributed inference architecture.

### 2.2. System Principles and Component Technologies

On-device inference at the racket is achieved through TinyML techniques, whereby the trained model is quantized, pruned, and optimized so that it can run directly on edge devices with limited computation and power [4]. The racket-side model is converted and deployed using STMicroelectronics’ STM32 X-CUBE-AI software package v.10.2.0, which automatically converts the trained network into optimized C code suitable for the STM32 MCU, with inference further accelerated via the CMSIS-NN library [11].

For inertial sensing, the system uses ICM-20948 nine-axis motion sensor, which integrates a three-axis accelerometer, a three-axis gyroscope, and a three-axis magnetometer [12]. As this study focuses on swing-motion features, magnetometer noise would add computational burden without providing additional benefit; only the accelerometer and gyroscope channels (six axes) are therefore used as model input features. Wireless communication on the racket side is handled by an HM-19 Bluetooth Low Energy (BLE 5.0) module built around a CC2640R2F radio MCU (Texas Instruments, Dallas, United States), configured via a UART interface using AT commands to simplify system integration.

Video acquisition uses an Allied Vision Mako G-131C PoE industrial camera (Stadtroda, Germany), equipped with a 1.3-megapixel (1280 × 1024) global-shutter CMOS sensor to avoid motion blur from fast swings, and supporting Gigabit Ethernet transmission and hardware-triggered GPIO for multi-device synchronization.

Two-dimensional pose estimation is performed using the open-source computer-vision tool Sports2D [13], which uses RTMPose-m as its pose-estimation backbone to predict joint coordinates for the head, shoulders, elbows, wrists, hips, knees, and ankles; temporal tracking maintains consistent subject identity across frames, and the resulting joint trajectories can be directly exported in a format compatible with OpenSim v. 4.5.

This study uses the open-source musculoskeletal modeling and biomechanical simulation platform OpenSim to convert two-dimensional pose trajectories into joint kinematic and kinetic features [14]. OpenSim represents the human body as a multi-body system of joints connected with specific degrees of freedom and uses inverse kinematics to estimate three-dimensional joint angles and corresponding joint moments, mapping motion information into a joint space that is biomechanically meaningful and easier to interpret.

The model architectures evaluated in this study included CNN, LSTM, CNN-LSTM, a Transformer encoder, and Random Forest. During preliminary screening on the three-class task, CNN and CNN-LSTM achieved the highest and most stable accuracy (83% and 80% for IMU-only, 81% and 81% for image-only, and 84% and 85% for multimodal, respectively), clearly outperforming LSTM, Transformer, and Random Forest (all ≤78% accuracy, with the Transformer’s fatigue-class recall as low as 22% in the multimodal setting); the remaining analysis therefore focuses on CNN and CNN-LSTM. CNN extracts local spatial or temporal patterns through convolution, an activation function (ReLU, Equation (1)), and pooling operations [15]; LSTM uses gating mechanisms and a cell state to retain information over long sequences while avoiding the vanishing/exploding gradient problems common in standard RNNs [16]. In the CNN-LSTM hybrid architecture, a CNN front end extracts local features from the raw multidimensional sequence, and the resulting feature sequence is then passed to an LSTM to model temporal dependencies, with final predictions produced by a Softmax classifier [17].

For reproducibility, the CNN consists of two convolutional layers (64 and 128 filters, kernel size 3, same padding) followed by batch normalization, global average pooling, and a dense layer (64 units, L2-regularized) before the softmax output, totaling 35,139 trainable parameters for the IMU input and 39,171 for the image input. The CNN-LSTM extends this architecture with a bidirectional LSTM layer (64 units per direction) inserted after the convolutional block and before global average pooling, totaling 133,955 parameters for the IMU input and 137,987 for the image input. Multimodal fusion is performed at the decision level via the weighted soft voting described in Section 2.3, rather than through a separate fused network architecture, and therefore introduces no additional trainable parameters beyond those of the two unimodal CNN-LSTM branches.

*f*(*x*) = *max*(0, *x*) (1)

### 2.3. Weighted Soft-Voting Decision Fusion

The decision-making process adopts soft voting, which combines the posterior class probabilities output by each model rather than using hard voting based on majority rule, thereby preserving each model’s prediction confidence [9]. Given N models and an input x, if *P_i_*(*y_j_|x*) denotes the probability assigned by model i to class j, the soft voting decision can be expressed as

(2) $$\hat{y}=arg\;max_{j}\left(1/N\right)\Sigma_{i=1}^{N}P_{i}\left(y_{j}|x\right)$$

Because the physiological evidence of fatigue captured by the IMU model and the vision model in this task is complementary, vision is better suited to identifying postural and compensatory changes, whereas the IMU signal is relatively more sensitive to local vibration during the transition stage, instead we adopted a weighted voting scheme. Letting the weight of each model be *w_i_*, with *Σ_i_*_=1_*^N^ w_i_* = 1, the weighted soft voting decision can be expressed as

(3) $$\hat{y}=arg\;max_{j}\Sigma_{i=1}^{N}w_{i}P_{i}\left(y_{j}|x\right)$$

The weight of each model is set in proportion to its overall F1-score on the validation set, giving better-performing models greater influence on the final decision. Taking the multimodal model’s weight as an example, it is calculated as

(4) $$w_{fusion}=M_{fusion}/\left(M_{fusion}+M_{IMU}+M_{Image}\right)$$

where *M_fusion_*, *M_IMU_*, and *M_Image_* denote the F1-scores of the multimodal, IMU-only, and image-only models on the validation set, respectively; the weights of the other two models are calculated in the same way, such that all weights sum to 1. Prior research indicates that this type of probability-weighted averaging can effectively reduce the impact of any single model’s estimation error on the overall decision [9]. Compared with hard voting, which discards prediction confidence altogether, retaining and weighting the confidence of each modality allows a high-confidence, high-weight model to dominate the final decision when another modality produces a low-confidence prediction due to noise or feature ambiguity, thereby improving recognition accuracy near the fatigue transition boundary.

### 2.4. Embedded AI Table Tennis Racket

**Figure 3:**
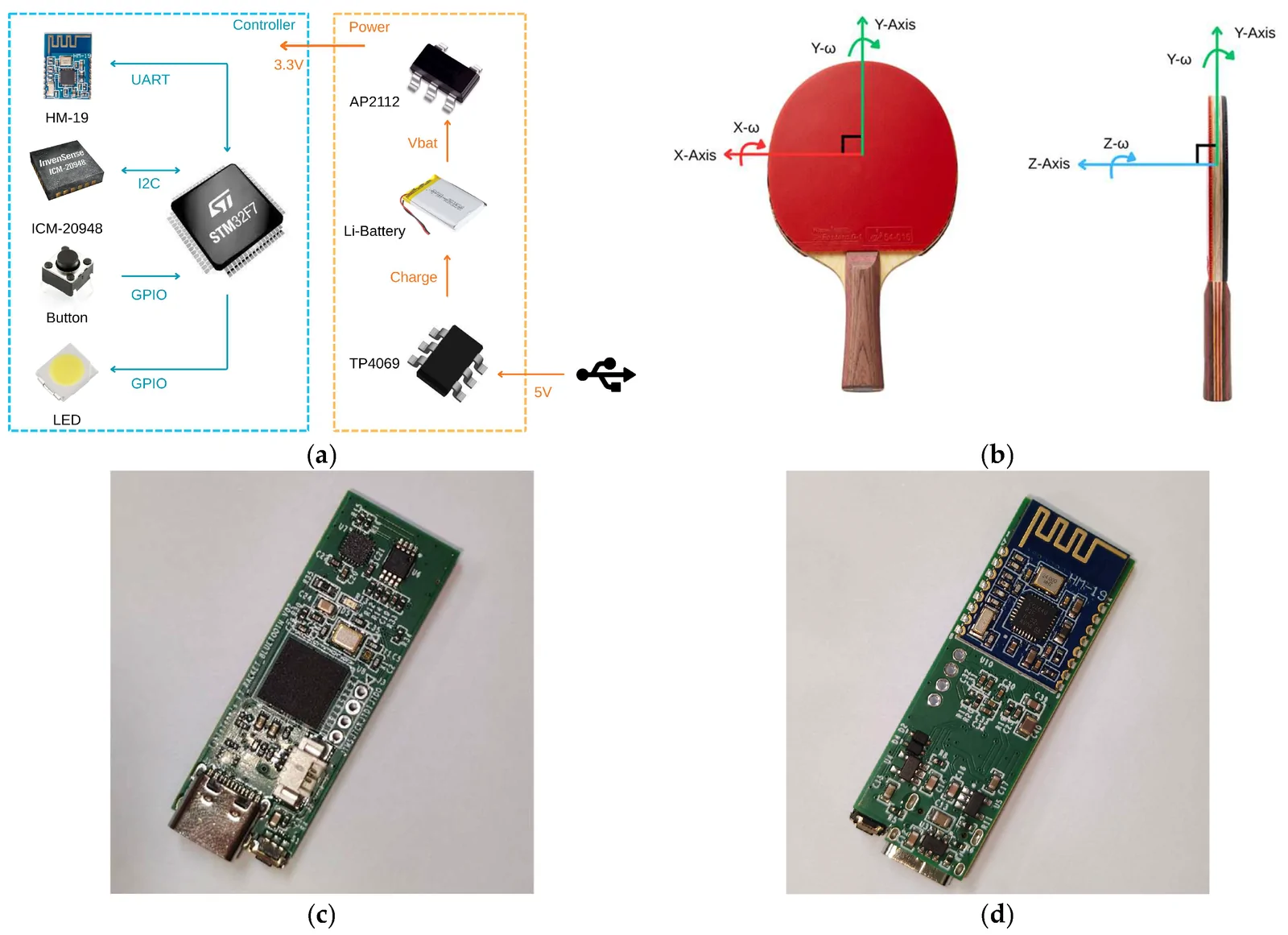
Embedded AI table tennis racket: (**a**) hardware architecture; (**b**) definition of the sensing axes relative to the racket face; (**c**) front view of the assembled circuit board; (**d**) back view of the assembled circuit board.

Figure 3a shows the hardware architecture of the embedded AI table tennis racket. The system uses an STM32F767 MCU as the main controller, responsible for peripheral communication control and on-racket TinyML inference. The racket integrates an HM-19 BLE module to wirelessly transmit sensor data and inference results to the host computer and is equipped with an ICM-20948 IMU to acquire acceleration and angular velocity data. Power is supplied by a rechargeable lithium battery, charged via a 5 V USB input, with an AP2112 regulator providing a stable 3.3 V output; a physical button and an LED indicator are also included for power control and status display. To preserve a natural grip feel, the circuit board is designed at 43 × 15 mm, allowing it to be embedded inside the racket handle without altering the player’s original grip. The handle was selected as the mounting location primarily to preserve a natural grip feel without altering the player’s original playing technique; the acquisition characteristics of alternative mounting positions (e.g., the racket blade or head), which may capture different vibration or impact signal profiles, were not empirically compared in this study and remain a direction for future work. As shown in Figure 3b, the racket’s three sensing axes are defined as follows: the red rubber side is the forehand face and the black rubber side is the backhand face; the Z-axis passes perpendicularly through the red rubber face, the X-axis runs horizontally along the red rubber face, and the Y-axis points upward along the handle.

The racket firmware’s finite-state machine is primarily responsible for sensor sampling, on-racket inference, and BLE communication. Once the Bluetooth connection is established, the firmware cycles through three states: (1) Idle state, in which it waits for a data collection command from the host. (2) Data collection and inference state, in which it continuously samples three-axis acceleration and angular velocity until the preset window size is reached, then performs preprocessing and executes on-racket inference; upon completion, it returns a “TT_OK” acknowledgment to the host and enters a waiting state. (3) Reply state, in which, upon receiving a result request from the host, it first returns the predicted class and its confidence value, then streams the complete frame-by-frame feature sequence used for that inference, before returning to the idle state to await the next cycle.

### 2.5. Data Preprocessing

This study applies preprocessing to the raw IMU signal: because table tennis strokes are fast and highly dynamic, the raw acceleration and angular velocity signals are susceptible to circuit noise and high-frequency vibration interference. A fourth-order Butterworth low-pass filter (sampling rate 85 Hz, cutoff frequency 5 Hz) is therefore applied to preserve the primary swing-motion signal while suppressing high-frequency noise unrelated to the motion, followed by five-point moving-average filtering to further smooth residual fluctuations.

This study uses Sports2D for pose estimation, with data augmentation enabled to improve robustness to occlusion and lighting variation, and the DeepSORT tracking mechanism employed to maintain consistent subject identity; detection is also restricted to the single person with the highest confidence value to avoid interference from background individuals (see Table 1).

The pose coordinates obtained from Sports2D were combined with the OpenCap markerless motion-capture pipeline [18] and converted, via OpenSim, into 18 joint kinematic features (angles and angular velocities) and 9 joint kinetic features (joint moments), for a total of 27 multimodal motion features. Videos of left-handed players are horizontally mirrored prior to processing to standardize the dominant-hand direction. Because the joint angles output by OpenSim can accumulate unbounded drift over time, which is detrimental to gradient-propagation stability and cross-subject generalization, all rotational features are re-encoded as sine–cosine pairs, mapping each angle θ to (sin θ, cos θ) within the bounded range [−1, 1]; this eliminates the discontinuity caused by angle wraparound while preserving the periodicity and directional information required to characterize joint dynamics [19]. As with the IMU signal, the biomechanical time-series data are also passed through a fourth-order Butterworth low-pass filter combined with moving-average filtering to suppress residual tremor arising from pose-estimation noise.

### 2.6. Multimodal Feature Alignment

Because the IMU (85 Hz) and image (60 Hz) data streams have different sampling rates, synchronization between the two devices was achieved at the software level: at the start of each test, the host computer issued a simultaneous start command that triggered data acquisition on both the racket-embedded IMU and the camera at the same instant. Temporal alignment for the multimodal model uses nearest-neighbor interpolation, with the higher-rate IMU stream serving as the reference timeline: each 60 Hz image feature is aligned to its nearest 85 Hz IMU timestamp, so that every time point simultaneously retains both the six-axis inertial features and the image-derived biomechanical features. Compared with linear interpolation, nearest-neighbor interpolation preserves the original sampled values and avoids generating interpolated features that lack physical meaning.

### 2.7. Participants and Data-Collection Protocol

**Figure 4:**
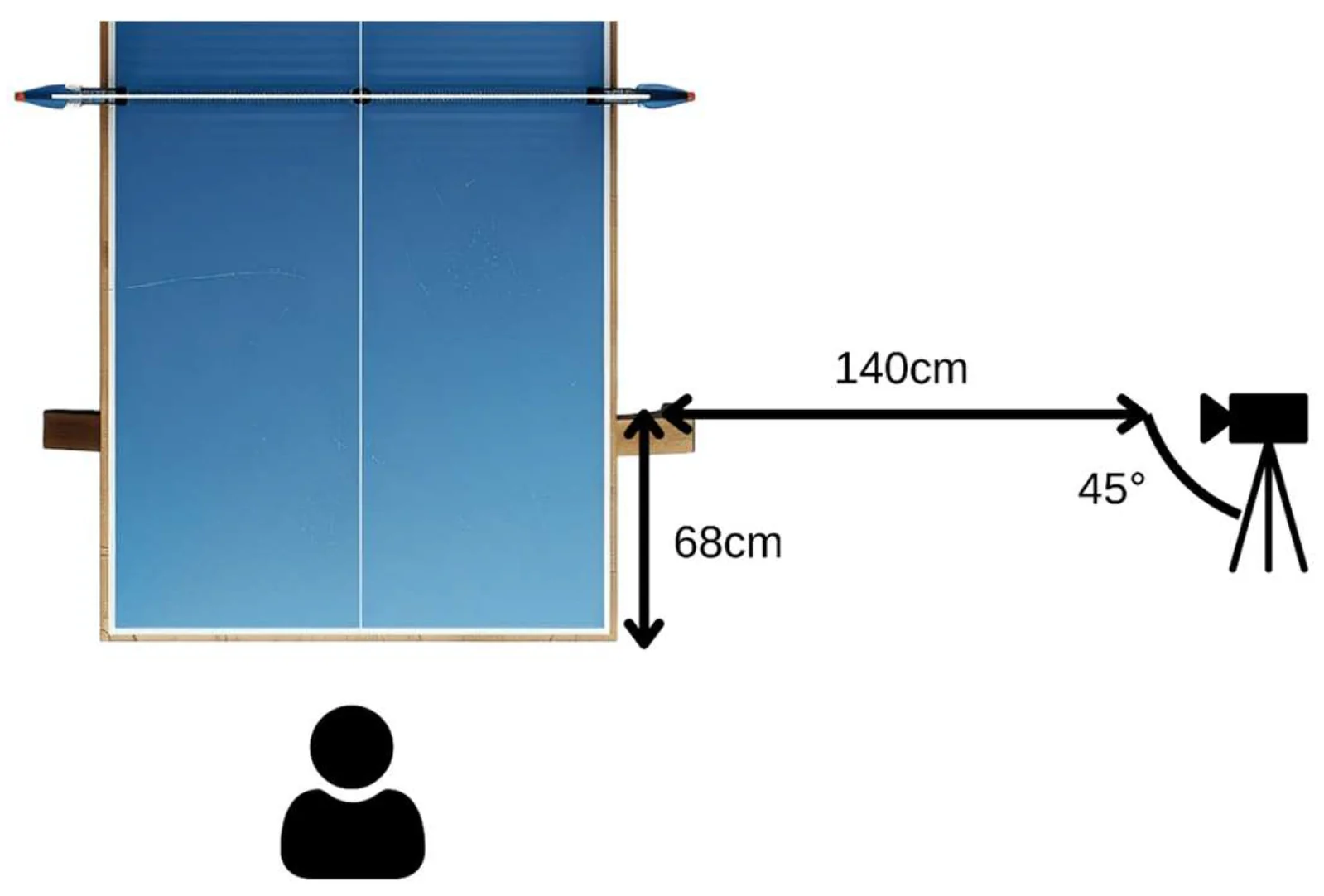
Camera placement used for stroke-motion video acquisition during aerobic hitting tests.

The camera configuration used in this study is shown in Figure 4. A total of 27 participants took part in this study, including university students and high-school table tennis players, and were tested using the embedded IMU racket. The data-collection procedure was approved by the Institutional Review Board (IRB) of Taichung Dali Jen-Ai Hospital, Taiwan (approval number: 202300074B0). All participants were fully informed of the study content and signed an informed consent form prior to data collection, and all data were collected, anonymized, and stored in accordance with the approved research ethics guidelines.

Participants were required to complete an incremental aerobic hitting test in forehand drive to simulate the progressive accumulation of fatigue during competition. The initial ball-feeding rate was set at 40 balls per minute, with the participant hitting continuously side to side; the feeding rate was then increased by 4 balls per minute every 3 min, progressively raising the exercise load, until the participant could no longer maintain the required hitting rhythm [20]. The test was terminated immediately if any of the following occurred: (i) the participant voluntarily requested to stop; (ii) the participant repeatedly failed to maintain the required hitting rhythm; (iii) the participant showed obvious signs of reduced coordination, delayed reaction, or postural instability; or (iv) the researchers judged, for safety reasons, that the test should not continue. These termination criteria were intended to induce functional exercise-related fatigue while preventing participants from being subjected to excessive physiological load.

The camera was positioned to the side of the table where the participant was located. Along the near baseline, its reference point was set 68 cm from the edge of the table toward the table’s midline (approximately the midpoint of the participant’s half of the table); from this point, the camera was placed 140 cm out from the side of the table (both measured horizontally on the ground plane), at a height of 75 cm above the ground, and rotated approximately 45° toward the participant to obtain a complete side view of the stroke. The camera configuration was also mirrored according to the participant’s dominant hand to ensure that key motion regions were consistently captured.

### 2.8. Fatigue-State Labelling and Classification Strategy

**Figure 5:**
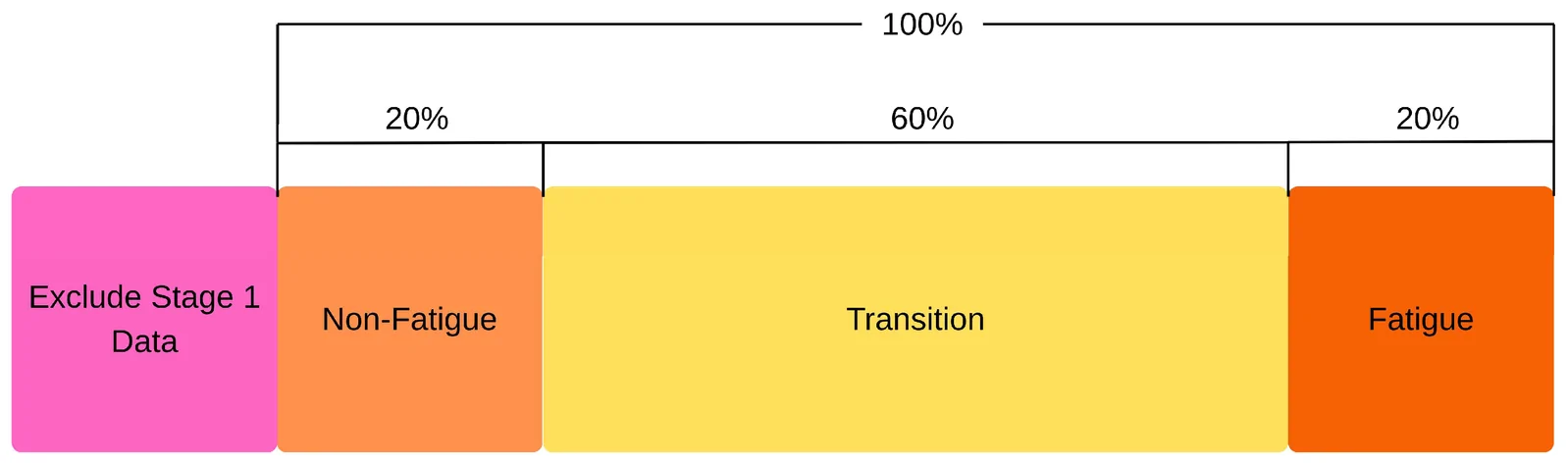
Feature-segmentation labelling scheme used to partition each trial into non-fatigue (first 20%), transition (middle 60%), and fatigue (final 20%) stages.

Because muscle fatigue in humans develops as a continuous physiological process rather than an instantaneous, discrete transition, this study adopts a segment-based labelling scheme that marks the data into three stages: non-fatigue, transition, and fatigue. According to cardiodynamic and oxygen-uptake kinetics theory, the early phase of exercise primarily reflects cardiac output and pulmonary blood-flow redistribution effects rather than steady-state skeletal-muscle and metabolic demand, with steady state typically reached after approximately 2–3 min [21]. Because each stage of the incremental ball-feeding protocol (Section 2.7) lasts exactly 3 min, data from the first complete stage, which falls within this theoretical transition window, were excluded during training to avoid distributional bias. As shown in Figure 5, after removing this initial phase, the remaining time-series data were divided chronologically into the first 20% (non-fatigue, a stable state in which energy supply and expenditure are balanced), the middle 60% (transition, reflecting the gradual accumulation of metabolic stress, declining neuromuscular efficiency, and increasing movement variability), and the final 20% (fatigue, corresponding to a marked rise in the slow component of oxygen uptake as it approaches VO_2_max).

This study adopts a progressive classification strategy: a binary classification task is used first, distinguishing only the two physiological states with the most pronounced difference (non-fatigue and fatigue) to verify the feasibility of recognition using IMU and image features under the most distinct physiological contrast. The transition stage is then incorporated to form a three-class classification task, allowing the model’s ability to track the continuous progression of fatigue under realistic training conditions to be evaluated. Samples with incomplete data or without sufficiently distinguishable motion features were removed during preprocessing. Because the proportion of such samples differs across modalities, and multimodal samples must additionally consist of fully paired IMU and image data collected from the same participant at the same time point, the number of usable samples is correspondingly smaller for the multimodal configuration than for either single modality; because transition stage samples (in the three-class task) account for 60% of all samples, a class-weighting mechanism was applied during training to mitigate class imbalance, and the data were split by participant into training and validation sets (80:20) to evaluate the model’s generalization to unseen participants. In total, across the 27 participants, preprocessing yielded 735 IMU windows (168 non-fatigue, 397 transition, 170 fatigue), 587 image windows (135, 314, and 138, respectively), and 545 multimodal windows (131, 301, and 113, respectively), corresponding to an average of approximately 27, 22, and 20 windows per participant for the IMU-only, image-only, and multimodal configurations.

### 2.9. Real-Time Fatigue-Recognition Software

The graphical real-time fatigue-recognition application is built using the Qt framework, with C++ (ISO/IEC 14882:2024) and Python v.3.14.8 jointly handling backend processing and model inference. Sensor communication, data processing, and model inference are each executed in background threads, ensuring that the interface remains responsive during real-time operation. The interface displays the live camera feed, the analysis status of the current test stage, and the fatigue classification result from the previous stage, and provides controls for device connection, camera activation, participant data entry, and test initiation.

During testing, the application simultaneously triggers the camera and the embedded racket, capturing a synchronized 3.5-s data window at each stage. The video stream is processed through Sports2D and OpenSim before being passed to the trained image model. The racket independently performs preprocessing of the IMU stream and on-racket inference, then transmits the features and prediction results back via Bluetooth. The host computer fuses the prediction results and features from both the image and IMU sides using the multimodal model and weighted soft voting to produce the fatigue classification result for that stage, which is displayed on the interface and simultaneously logged to a CSV file. This cycle continues until the test ends, and the system plots a time-series curve of the fatigue classification results across stages to show the fatigue-progression trend for that test session.

## 3. Results and Discussion

### 3.1. Model Training Configuration

All models were trained using a unified training configuration, as shown in Table 2. A default 2.5-s sliding window with a 0.5-s stride was used, with a feature sampling rate of 85 Hz (IMU and multimodal) or 60 Hz (image). Models were trained using the Adam optimizer for up to 200 epochs, with early stopping and a learning-rate-on-plateau reduction mechanism. Dropout, L2 regularization, and label smoothing were applied during training to reduce model overconfidence, and Scikit-learn’s balanced class weighting was used to offset the effect of the larger number of transition-stage samples.

### 3.2. Binary Fatigue Classification Performance

**Figure 6:**
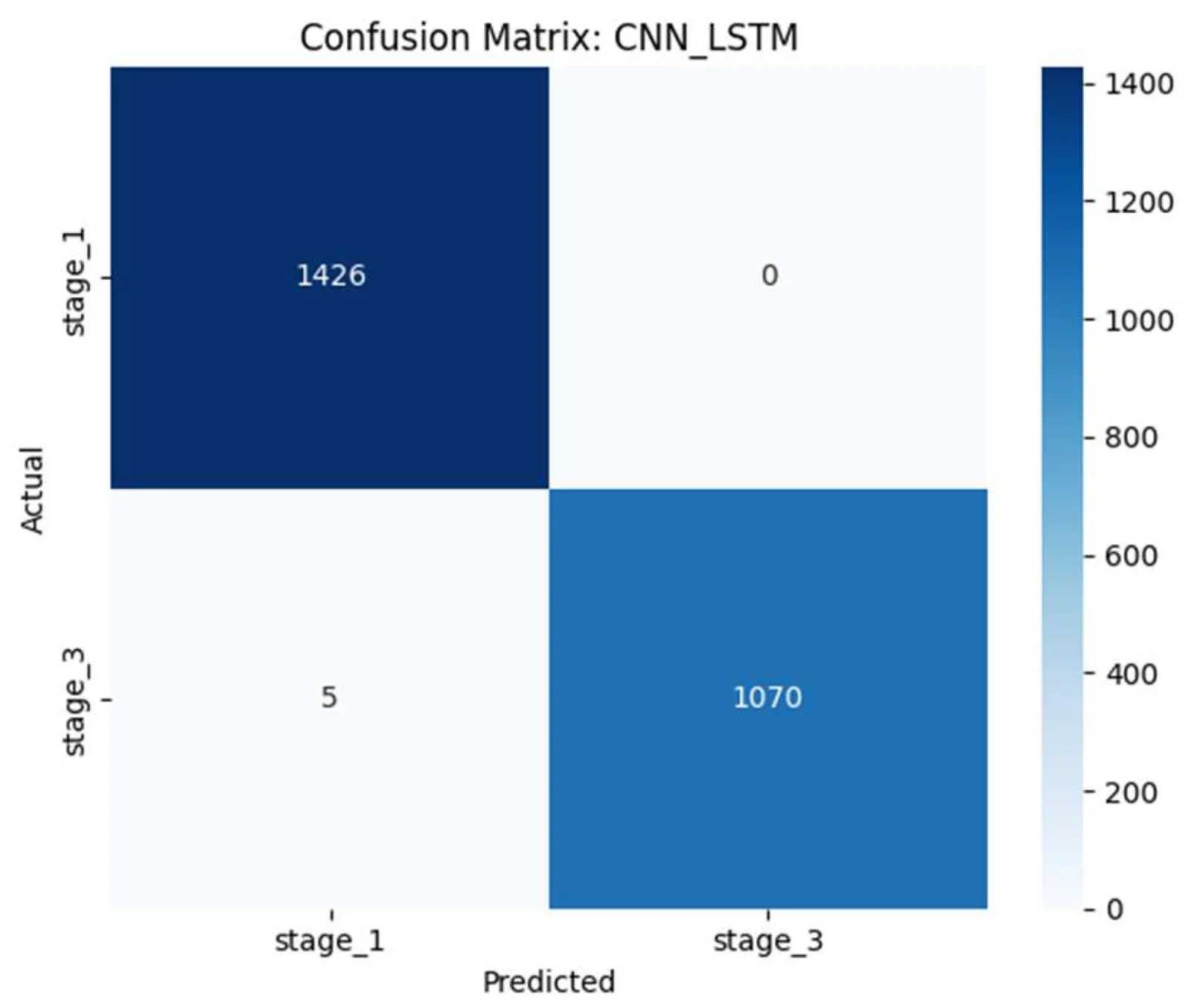
Confusion matrix of the CNN-LSTM model on the validation set for the binary (2-class) multimodal task.

This study first evaluated the performance of two architectures, CNN and CNN-LSTM, on the binary classification task (non-fatigue, fatigue) under three sensing modalities (IMU only, image only, and multimodal) to verify the feasibility of recognition under the most physiologically distinct extreme states. The results show that high accuracy was achieved across all modalities on the binary task: 95–96% for IMU only, 97% for image only, and 99% for multimodal fusion (see Table 3). As shown in Figure 6, in the confusion matrix of the multimodal CNN-LSTM model, non-fatigue samples were classified with zero errors, with only 5 fatigue samples misclassified. This result confirms that the sensing and feature-extraction architecture used in this study is already approaching a performance ceiling when distinguishing two clearly separated physiological states, motivating the subsequent inclusion of the transition stage to evaluate the model’s ability to recognize continuous fatigue change under conditions closer to actual training scenarios.

### 3.3. Three-Class Fatigue Classification Performance

As shown in Table 4, this study compared the performance of CNN and CNN-LSTM on the three-class classification task (non-fatigue, transition, fatigue) across three sensing modalities (IMU, image, and multimodal), under two window lengths, 2.5 s and 3.5 s. With the default 2.5-s window, multimodal fusion achieved the highest accuracy (84% for CNN, 85% for CNN-LSTM), outperforming IMU only (83% for CNN, 80% for CNN-LSTM) and image only (81% for both CNN and CNN-LSTM); the confusion matrix of the IMU model showed that most misclassifications occurred between adjacent fatigue stages, consistent with existing HAR research findings that brief transitional movements between two clearly separated states tend to produce unstable predictions [22]. Because a trade-off exists between feature completeness and system latency, this study further re-evaluated the models using a 3.5-s window in place of the default 2.5-s window: the IMU model benefited from the longer window, with CNN-LSTM accuracy increasing from 80% to 82% and fatigue recall rising from 84% to 87%, as the longer window captures a more complete backswing-to-stroke cycle; the image model’s performance declined with the longer window, as it introduced more uninformative recovery-posture frames, with CNN-LSTM fatigue recall dropping from 88% to 74%; the multimodal model, able to compensate for the degradation on the image side using IMU information, showed a stable improvement to 87% accuracy for both architectures at 3.5 s.

### 3.4. Effect of Weighted Soft Voting

**Figure 7:**
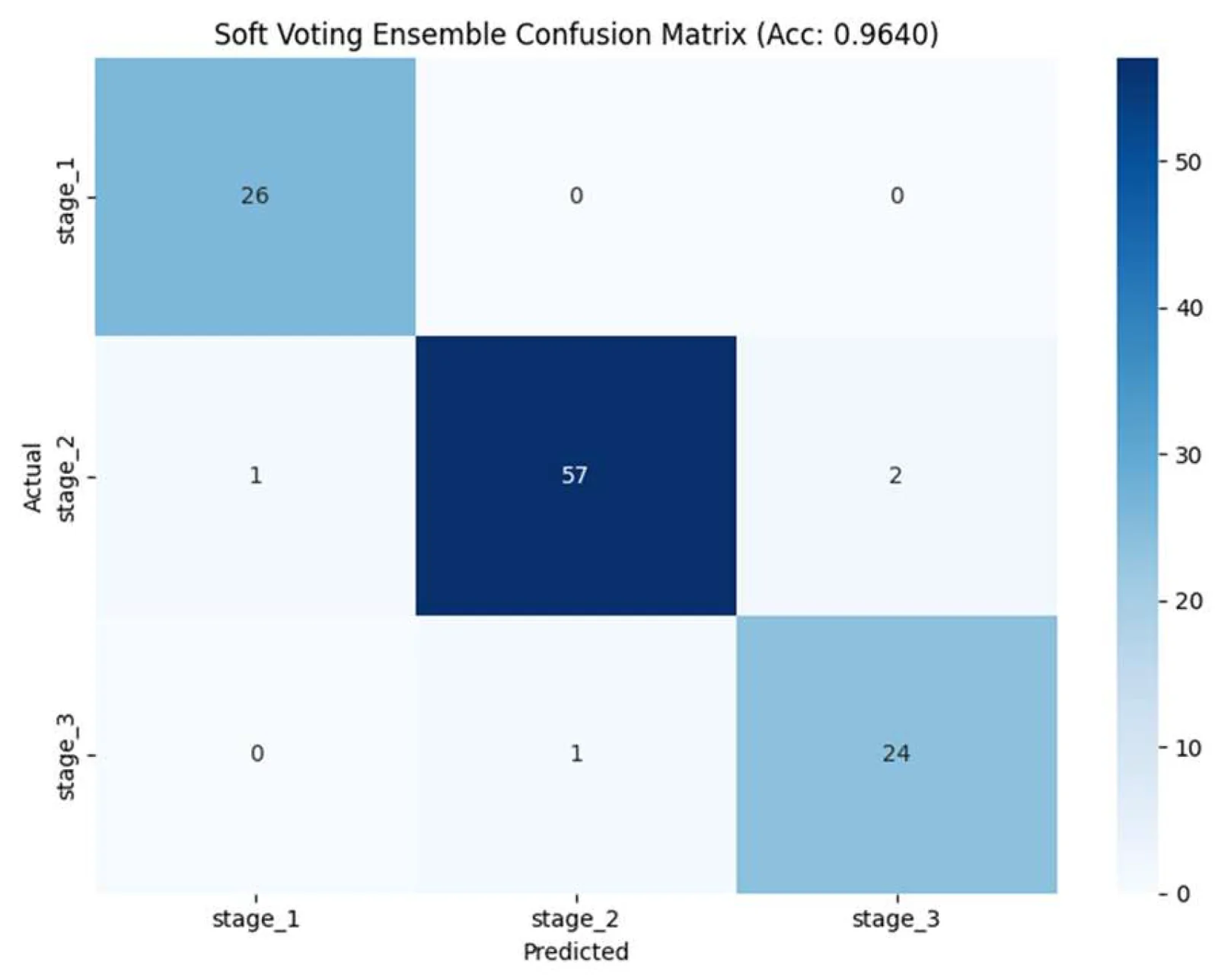
Confusion matrix on the validation set after F1-score-weighted soft voting across the IMU, image and multimodal models (three-class task, 3.5 s window).

Using the previously identified better-performing 3.5-s window CNN-LSTM configuration, F1-score-weighted soft voting was applied to the output results of the IMU, image, and multimodal models, the overall three-class accuracy improved to 96 (Table 5). As shown in Figure 7, the remaining misclassifications were concentrated mainly between adjacent fatigue stages, indicating that weighted decision fusion, by integrating the complementary misclassification patterns of the different modalities, can substantially improve recognition accuracy near the transition boundary.

### 3.5. Embedded Feasibility and On-Device Latency

This study used STM32 X-CUBE-AI to convert the IMU model into a form executable on the racket in Table 6. The resulting executable requires approximately 549.54 KB of read-only (Flash) memory and 153.91 KB of RAM, confirming that the model satisfies the resource constraints of the target MCU and that the racket can perform edge AI inference within these constraints. The target STM32F767 MCU provides 512 KB of SRAM; the deployed model’s 153.91 KB footprint therefore occupies approximately 30% of available RAM, leaving sufficient margin for the sensor-data buffer and firmware overhead. In addition, the racket’s 150 mAh battery supports approximately 60 min of continuous operation from a full charge; given an average complete inference cycle time of 60.70 s (Table 7), a single full charge therefore supports approximately 59 consecutive inference cycles (3600 s/60.70 s ≈ 59), corresponding to roughly 60 min of continuous fatigue monitoring, which is more than sufficient to cover a complete incremental hitting-test session under this study’s protocol, in which the exercise stage changes every 3 min. End-to-end communication between the host computer and the embedded racket was verified using a dedicated test program: a “TT_OK” acknowledgment was correctly received after each data-collection/inference command, and the classification result, confidence value, and streamed feature data were correctly returned after each result-request command; repeated testing showed no packet loss or data anomalies, confirming that the system provides stable data transmission during real-time operation.

### 3.6. Real-Time System Performance

**Figure 8:**
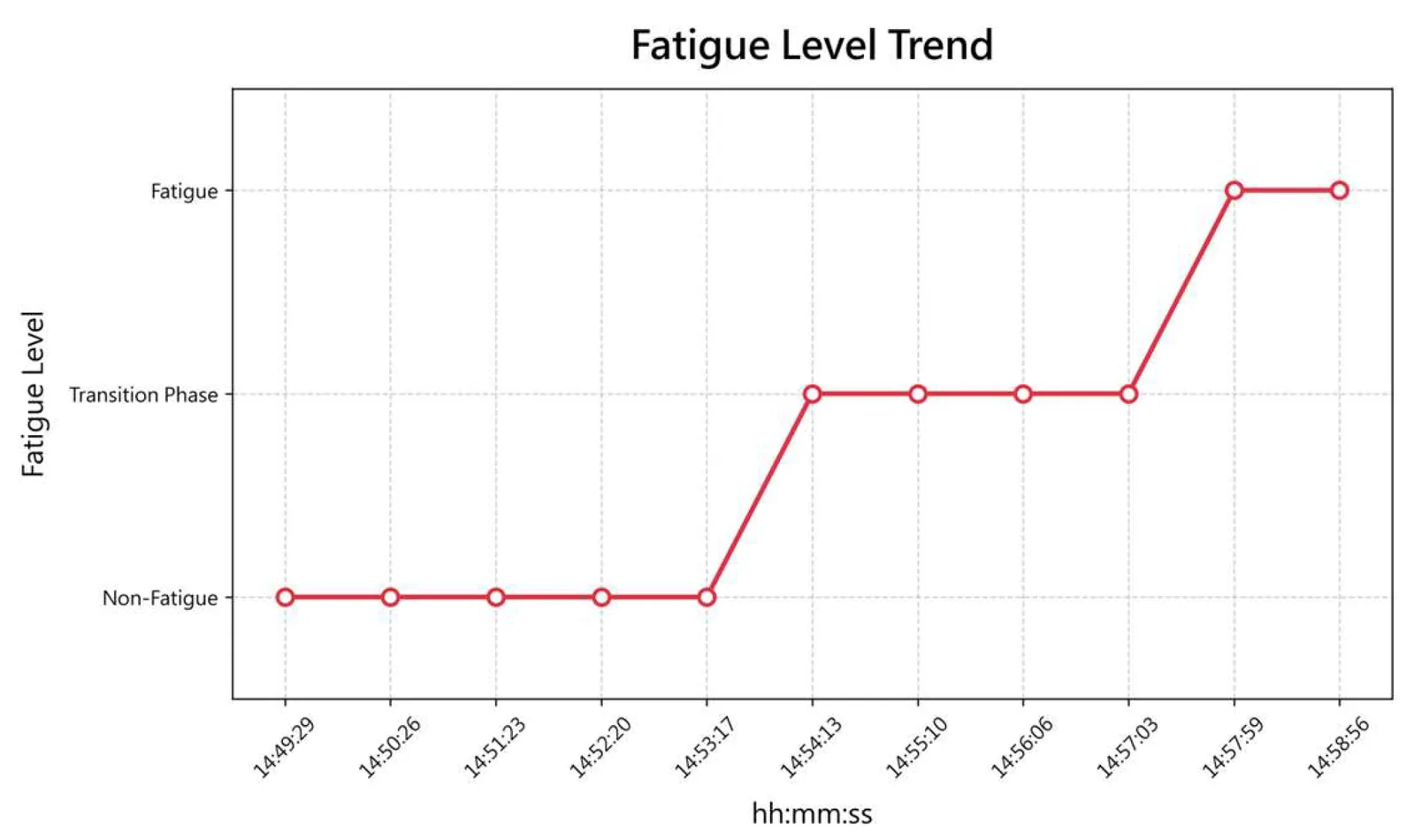
Representative output of the real-time fatigue-recognition system displayed in the host-computer interface.

As shown in Figure 8, the complete pipeline comprises: device connection handshake, camera and racket initialization, synchronized 3.5-s data acquisition, on-racket inference, host-side Sports2D/OpenSim processing, multimodal fusion, and weighted-voting output was verified end to end and correctly displayed in the graphical interface, with results from each stage logged for subsequent fatigue-curve visualization.

This study timed ten complete inference cycles: the racket-side pipeline took an average of 29.04 s, the image-side pipeline (Sports2D/OpenSim processing and inference) took an average of 51.63 s, and the overall average cycle time was 60.70 s (Table 7), indicating that the main source of system latency is the pose-estimation and biomechanical conversion pipeline rather than data transmission or model inference itself. Because the experimental protocol advances to the next exercise stage every 3 min, the system’s roughly one-minute cycle time allows up to three fatigue classification updates per stage, meeting the timing requirements of the test protocol. This demonstrates that the computational efficiency of the proposed system is sufficient to meet the timing requirements of the experimental protocol, enabling continuous, stage-wise fatigue assessment throughout the test, rather than a single post-hoc judgement made only after testing is complete.

## 4. Conclusions

This study makes three main conclusions. First, it demonstrates a dual-modality fatigue-monitoring framework combining an embedded IMU racket with vision-based pose estimation, fused through weighted soft-voting decision fusion, that is feasible and computationally lightweight for use during table tennis training. Second, by dividing fatigue accumulation into three interpretable stages, the framework distinguished the two most extreme states with 99% accuracy in a binary formulation and reached 84–87% accuracy in a three-class formulation that improved to 96% after multimodal fusion, indicating that multimodal fusion may particularly aid discrimination during the transition between non-fatigue and fatigue, where single-modality models appear more prone to misclassification. Third, embedding the circuit board within the racket handle preserves the player’s natural grip while showing that on-device inference is achievable within the memory and computational limits of a low-power microcontroller without a persistent network connection, with an overall cycle time adequate relative to the 3-min interval between training-load changes; this latency appears to arise mainly from host-side pose estimation and biomechanical conversion rather than on-device inference, pointing to the image-processing pipeline as the priority target for future latency reduction.

Nevertheless, three limitations should be noted. First, fatigue stages were defined by a time-based segmentation grounded in physiological theory rather than direct per-stage physiological measurement, and because the ball-feeding rate increased on the same schedule as the stage transitions, the labels are not fully independent of elapsed time and hitting frequency; the feature-extraction windows also overlapped between adjacent windows, which should be considered when interpreting the reported sample sizes and performance metrics. Second, three-dimensional kinematic and kinetic features were derived from single-camera pose estimation combined with a generic musculoskeletal model, whose accuracy relative to multi-camera or personalized modeling was not independently validated in this study. Finally, the IMU was mounted at a single location on the racket handle, so applicability to other sensor placements remains to be clarified.

Future work will focus on five directions: reducing image-processing latency through model lightweighting or dedicated hardware acceleration; verifying the reported performance using a participant-grouped nested cross-validation scheme; incorporating additional physiological indicators, including continuous fatigue quantification, to refine the fatigue labels; cross-checking the biomechanical pipeline against multi-camera or marker-based systems across additional IMU placements and stroke types; and extending data collection to a broader range of ages, sexes, and skill levels, cross-validated against existing assessment tools, to further examine generalizability.

## Figures and Tables

**Table 1:** Sports2D configuration parameters.

Parameter (Flag)	Value
Data augmentation: --use_augmentation	True
Tracking mode: --tracking_mode	DeepSORT
Number of persons to detect: --nb_persons_to_detect	1
Person-ordering method: --person_ordering_method	Highest likelihood

**Table 2:** Model training parameter configuration.

Category	Parameter	Value
Feature processing	Sampling rate (FS)	IMU: 85 Hz Image: 60 Hz Multimodal: 85 Hz
	Window size	2.5 s
	Stride	0.5 s
Training	Optimizer	Adam (LR = 1 × 10^−4^)
	Batch size	32
	Epochs	200
	Early stopping	Patience = 15
	LR reduction on plateau	Factor = 0.5, patience = 5
Regularization	Dropout	0.3
	L2 regularization	0.01
	Label smoothing	0.1
Class balancing	Class weight	Balanced

**Table 3:** Binary (2-class) classification accuracy for CNN and CNN-LSTM across sensing modalities.

Modality	Model	Accuracy
IMU	CNN	96%
IMU	CNN-LSTM	95%
Image	CNN	97%
Image	CNN-LSTM	97%
Multimodal	CNN	99%
Multimodal	CNN-LSTM	99%

**Table 4:** Three-class classification accuracy and fatigue-class recall for CNN and CNN-LSTM across sensing modalities and window lengths.

Modality	Model	Acc. 2.5 s	Fatigue Recall 2.5 s	Acc. 3.5 s	Fatigue Recall 3.5 s
IMU	CNN	83%	80%	83%	75%
IMU	CNN-LSTM	80%	84%	82%	87%
Image	CNN	81%	67%	80%	69%
Image	CNN-LSTM	81%	88%	81%	74%
Multimodal	CNN	84%	73%	87%	82%
Multimodal	CNN-LSTM	85%	82%	87%	82%

**Table 5:** Classification performance on the validation set after F1-score-weighted soft voting (three-class task, 3.5 s window).

Class	Precision	Recall	F1-Score	Accuracy
Non-fatigue (stage_1)	96%	100%	98%	96%
Transition (stage_2)	98%	95%	96%	
Fatigue (stage_3)	92%	96%	94%	

**Table 6:** STM32 X-CUBE-AI memory-footprint analysis for the on-device IMU model.

Item	Size
Total Flash	549.54 KiB (562,724 B)
Weights	521.01 KiB
Library	28.52 KiB
Total RAM	153.91 KiB (157,608 B)
Activations (incl. Input 6.96 KiB)	149.75 KiB

**Table 7:** Per-module processing time over ten complete real-time inference cycles.

Cycle	Racket Time (s)	Image Time (s)	Total Time (s)
1	29.062	51.394	60.505
2	29.071	50.864	59.936
3	29.048	50.922	59.958
4	29.060	50.877	60.016
5	29.054	50.674	59.702
6	29.009	51.565	60.654
7	29.035	51.855	60.913
8	29.008	52.864	61.924
9	29.016	52.699	61.766
10	29.030	52.580	61.645
Mean	29.039	51.629	60.702

## Data Availability

The raw data supporting the conclusions of this article will be made available by the authors on reasonable request.
